# SMS-based family planning communication and its association with modern contraception and maternal healthcare use in selected low-middle-income countries

**DOI:** 10.1186/s12911-020-01228-5

**Published:** 2020-09-10

**Authors:** Yingying Hu, Rui Huang, Bishwajit Ghose, Shangfeng Tang

**Affiliations:** 1 The Department of Obstetrics and Gynecology, The Fourth Affiliated Hospital, Zhejiang University School of Medicine, Yiwu, Zhejiang, 322000 China; 2 School of Pharmacy, Tongji Medical College, Huazhong University of Science and Technology, Wuhan, 430030 China; 3 School of Medicine and Health Management, Tongji Medical College, Huazhong University of Science and Technology, Wuhan, China

**Keywords:** Family planning, SMS communication, Maternal healthcare services, Demographic and health survey

## Abstract

**Background:**

The objectives of this study were to 1) measure the percentage of women who received SMS-based family planning communication, and 2) its association with modern contraception and maternal healthcare services among mothers. In recent years, there has been a growing interest surrounding mobile phone-based health communication and service delivery methods especially in the areas of family planning and reproductive health. However, little is known regarding the role of SMS-based family planning communication on the utilisation of modern contraception and maternal healthcare services in low-resource settings.

**Methods:**

Cross-sectional data on 94,675 mothers (15–49 years) were collected from the latest Demographic and Health Surveys in 14 low-and-middle-income countries. The outcome variables were self-reported use of modern contraception and basic maternal healthcare services (timely and adequate use of antenatal care, and of facility delivery services). Data were analysed using multivariate regression and random effect meta-analyses.

**Results:**

The coverage of SMS-based family planning communication for the pooled sample was 5.4% (95%CI = 3.71, 7.21), and was slightly higher in Africa (6.04, 95%CI = 3.38, 8.70) compared with Asia (5.23, 95%CI = 1.60, 8.86). Among the countries from sub-Saharan Africa, Malawi (11.92, 95%CI = 11.17, 12.70) had the highest percent of receiving SMS while Senegal (1.24, 95%CI = 1.00, 1.53) had the lowest. In the multivariate analysis, SMS communication shown significant association with the use of facility delivery only (2.22 (95%CI = 1.95, 2.83). The strength of the association was highest for Senegal (*OR* = 4.70, 95%CI = 1.14, 7.33) and lowest for Burundi (*OR* = 1.5; 95%CI = 1.01, 2.74). Meta analyses revealed moderate heterogeneity both in the prevalence and the association between SMS communication and the utilisation of facility delivery.

**Conclusion:**

Although positively associated with using facility delivery services, receiving SMS on family planning does not appear to affect modern contraceptive use and other components of maternal healthcare services such as timely and adequate utilisation of antenatal care.

## Background

Healthcare systems in the LMICs have been struggling to promote contraceptive prevalence rates to reduce the burden of wanted pregnancy and unmet need for family planning and improving maternal and child survival. Given women’s rising age of marriage and increasing labour force participation with concurrent changes in fertility preferences in the low-middle-income countries (LMICs) [1–3], the need for making family planning services universal and accessible to all eligible women in an equitable manner is higher than ever [4–6].

Apart from the direct contraception related services, for a great proportion of women family planning centres also serve as the main interface with healthcare providers and thereby offers the opportunity to enhance not only of family planning services, but also the essential maternal healthcare services such as prenatal care and skilled birth services [7]. Non- and underutilisation of maternal healthcare services and associated maternal morbidities and mortalities constitute another serious public health challenge in most low-middle-income countries [8–12]. This way, family planning has a key role to play in promoting overall sexual and reproductive health and achieving maternal health related SDGs [4, 5]. The well-documented advantages notwithstanding, worldwide a large proportion of the women of reproductive age remain deprived of basic family planning services, especially among the medically underserved communities in the LMICs [7]. In 2015 for instance, the prevalence of contraceptive use in sub-Saharan Africa was 24% compared with the least developed country average of 34% and the global average of 57% [13]. Persistent barriers to promoting contraceptive prevalence rate underscore the need for developing more equitable and long-lasting intervention strategies in a cost-effective manner.

Promotion of family planning services in LMICs are generally hindered by various political, financial, skilled health professional and infrastructural constraints [14–16]. With the continued progress in the technological sector and its growing collaboration with healthcare systems, however, researchers are being able to engineer innovative solutions to the long-standing infrastructural issues in the provision of health services, including the discipline of sexual and reproductive healthcare. For instance, the application of telemedicine and SMS-based (Short message service) health communication services are facilitating services ranging from as basic as teaching reproductive health to school students [17] to services as complex as abortion [18–20]. The rapid of expansion of mobile phone ownership have opened avenues for patient monitoring [21, 22] and providing services to disadvantaged population especially in the remote areas where service availability is sparse [23–25]. Although comparatively less common, mobile health-based family planning services such as contraceptive counselling, menstrual regulation [12], selection of methods, reminder messages, implant and providing support for side-effects are becoming increasingly popular [26, 27].

Given the rising penetration of telecommunication market and increasing number of subscribers, the prospect of SMS-based family planning interventions as cost-effective alternative to clinical visits remain high, particularly for people living in the remote areas. Several protocol studies in LMICs e.g. Cambodia [27], Bangladesh [28], Bolivia [29], Tajikistan [30], are being carried out in the context of mobile-based health interventions. However, data on the effects of SMS-based family planning services on contraceptive use and uptake of MHS are still very limited, especially in the LMICs. To this regard, we undertook the present study using nationally-representative data from 14 LMICs: Angola, Armenia, Burundi, Ethiopia, Haiti, Malawi, Nepal, Philippines, Senegal, Tanzania, Timor Leste, Senegal, Uganda and Zimbabwe. Using cross-sectional data from the latest Demographic and Health Surveys on these countries, our aim was to measure the prevalence of mothers receiving SMS regarding family planning, as well as the association between receiving SMS with modern contraceptive use, and utilisation of essential maternal health services. Selection of the countries was based on the availability of data on receiving SMS and use of maternal health services. Details of the surveys and measurement of the variables are presented in the following section.

## Methods

### Settings

The scope of the study was 14 LMICs, including nine from sub-Saharan Africa (Angola, Burundi, Ethiopia, Malawi, Senegal, Tanzania, Senegal, Uganda and Zimbabwe), four from Asia (Armenia, Nepal, Philippines, Timor Leste), and one from North America (Haiti). Details of the countries and their respective survey implementing bodies were presented in Table 1.

**Table 1:** Description of the surveys included in the study

Country	Year of survey	Interviewed	Response Rate	Implementing body
Angola	2015/16	14,379	96%	Estratégia Nacional de Desenvolvimento Estatístico (ENDE)
Armenia	2015/16	6116	97.8	National Statistical Service (NSS) and the Ministry of Health (MOH)
Burundi	2016/17	17,269	98.8	l’Institut de Statistiques et d’Études Économiques du Burundi (ISTEEBU)
Ethiopia	2016	15,643	98.6	Central Statistical Agency (CSA)
Haiti	2016/17	14,371	98.9	l’Institut Haïtien de l’Enfance (IHE)
Malawi	2015/16	24,562	97.7	National Statistical Office
Nepal	2016	12,862	98.3	New ERA
Philippines	2017	27,496	98.7	Philippine Statistics Authority (PSA)
Senegal	2017	8865	95.9	l’Agence Nationale de la Statistique et de la Démographie (ANSD).
Timor Leste	2016	12,607	97	General Directorate of Statistics, Ministry of Planning and Finance, Ministry of Health
Tanzania	2015/16	13,266	97	National Bureau of Statistics (NBS) and Office of the Chief Government Statistician (OCGS)
Uganda	2016	18,506	97	Uganda Bureau of Statistics.
Zimbabwe	2015	9955	96.2	Zimbabwe National Statistics Agency

### Data source

Data used in this study are sourced from the Demographic and Health Surveys program. These surveys are nationally representative and collect data on a range of demographic, socioeconomic, and maternal health and family planning related indicators by host countries with financial support by USAIDS and technical assistance by ICF international. Demographic and Health Surveys operates in about 90 LMICs with the primary objective of providing quality data on health and development indicators to facilitate evidence-based policy making and monitoring of development programmes. Out of initial 248,186 participants in the pooled sample, respectively 46,297 (never experienced pregnancy), 14,706 (no formal education) and 92,508 (owns no phone) were removed from the analysis and the remaining 94,675 were included for analysis. For this study, the sample population were mothers of childbearing age (15–49 years). Further details of the surveys are shown in Table 1, and are available through Demographic and Health Surveys manuals.

### Measures

The outcome variables of this study were modern contraceptive use and maternal healthcare services including antenatal care and place of childbirth. Contraceptive use was measured by asking the type of methods participants are currently using. The answers were: Not using, using traditional method, [31] and using modern methods. Those who reported using modern methods were categorised as “Yes”, and the rest as “No”.

For antenatal care, both the timing and number of visits were analysed. Timing of initiation of ANC was categorised as “Timely” (within 1st trimester) and “Late” (after 1st trimester) [12]. Frequency of ANC visits was categorised as “Adequate” (at least 4 visits) and “Inadequate” (< 4 visits) [32].

Place of childbirth was categorised as “Home” (Home of respondents/relatives) and “Facility” (hospital, clinic, other health centres) [9].

The main independent variable of interest was receiving family planning related SMS among the participants. Respondents were asked whether or not they received any text-message regarding FP, and the answers were classified as “Yes” and “No”.

In order to adjust the analysis for potential confounders, several demographic and socioeconomic factors were entered into the analysis as well: Age groups (15–19/ 20–24/ 25–29/ 30–34/ 35–39/ 40–44/ 45–49); Residence (Urban/ Rural); Occupation (Not working/ Agri-domestic/ Professional-technical); Wealth quintile [33] (Q1/Lowest Q2/ Q3/ Q4/ Q5/Highest); Parity (1–2/ 3–4/ > 4) Reads newspaper (No/ Yes); Listens to radio (No/ Yes); Watches TV (No/ Yes) [34–38].

### Data analysis

Data analyses were performed with STATA version 14 and SPSS version 24. The datasets first cleaned to retain the observations that met the selection criteria, and were checked for multicollinearity among independent variables. Datasets then merged to perform pooled analysis. As the survey used multistage sampling techniques, we used complex analysis accounting for the primary sampling units, sample strata, and weight, as recommended for Demographic and Health Surveys surveys [39]. Descriptive analyses were carried out to present the sociodemographic characteristics of the sample population and are shown as percentages. Bar charts were used to show the prevalence of modern contraceptive use and utilisation of maternal health services relative to receiving FP messages. Meta-analysis of the 14 countries was conducted to show the percentages (with 95%CI) of receiving FP related SMS as forest plot. To measure the multivariate odds of association between receiving FP related SMS with the outcome variables, we used binary logistic regression analyses. Five sets of regression analyses were run to adjust the association for variables of interest at each step. The level of significance was set at *p* < 0.05. For Sensitivity test, we performed sub-group analyses for each of the 14 countries to assess whether the association varied from the pooled analysis.

### Ethics statement

Demographic and Health Surveys are approved by ICF international, USA. All participants gave informed consent before taking part in the survey. Data are available in the public domain in anonymised form, therefore no additional approval was necessary.

## Results

### Descriptive results

Basic sociodemographic characteristics of the sample population were presented in Table 2. In short, a larger proportion of the mothers were in the age group of 30–34 years (20.2%), of rural residence (52.4%), had professional/technical employment (40.7%), from highest wealth index households (25.9%), had 1–2 children (51.4%), not used to read newspaper (61.7%), used to listen to radio (61.8%) and watched TV (75.2%).

**Table 2:** Description of the sample population (*n* = 94,675)

Variables	Overall (%)	Modern Contraceptive (45.14%)	Timely ANC (61.25%)	Adequate ANC (76.92%)	Facility delivery (75.68%)
**Age groups**					
15–19	2.6	2.1	3.9	4.1	4.5
20–24	13.3	13.0	20.8	21.6	22.1
25–29	19.9	20.7	29.1	28.6	28.1
30–34	20.2	21.8	23.9	23.5	23.3
35–39	17.8	19.6	14.6	14.5	14.4
40–44	14.5	14.6	6.4	6.3	6.2
45–49	11.7	8.3	1.3	1.4	1.4
*p-value*		*<.001*	*<.001*	*<.001*	*<.001*
**Residency**					
Urban	47.6	48.1	49.7	49.2	52.5
Rural	52.4	51.9	50.3	50.8	47.5
*p*-value		<.001	<.001	<.001	<.001
**Occupation**					
Not working	33.2	33.4	41.0	40.4	38.9
Agri/domestic	26.0	25.1	19.9	21.3	23.0
Professional/technical	40.7	41.5	39.1	38.3	38.1
*p-value*		*<.051*	*<.104*	*<.021*	*<.001*
**Wealth status**					
Q1 (Lowest)	15.8	15.5	15.8	14.9	10.1
Q2	17.3	17.5	17.2	17.1	15.1
Q3	19.4	18.8	19.6	19.7	19.5
Q4	21.6	21.6	21.6	22.2	24.1
Q5 (Highest)	25.9	26.6	25.8	26.2	31.1
*p-value*		*<.001*	*<.001*	*<.001*	*<.001*
**Parity**					
1–2	51.4	50.6	60.3	58.2	57.7
3–4	30.8	33.9	27.3	27.9	27.6
> 4	17.9	15.5	12.4	13.9	14.7
*p-value*		*<.001*	*<.001*	*<.001*	*<.001*
**Reads newspaper**					
No	61.7	57.3	58.0	58.6	59.4
Yes	38.3	42.7	42.0	41.4	40.6
*p-value*		*<.001*	*<.001*	*<.001*	*< 0.401*
**Listens to radio**					
No	38.2	38.1	36.5	35.9	32.6
Yes	61.8	61.9	63.5	64.1	67.4
*p-value*		*<.001*	*<.001*	*<.001*	*<.001*
**Watches TV**					
No	24.8	20.4	20.3	23.2	26.8
Yes	75.2	79.6	79.7	76.8	73.2
*p-value*		*<.001*	*<.001*	*<.001*	*<.001*

Table 2 also indicates the prevalence of using modern contraceptive (45.14%), timely ANC (61.25%), Adequate ANC (76.92%), and Facility delivery (75.68%). Significant demographic and socioeconomic patterns were observed in the prevalence of all four outcomes. Prevalence of modern contraceptive use was highest in the age group of 30–34 years (21.8%), whereas that of timely ANC contact, adequate ANC visits and facility delivery was highest among those aged 25–29 years. Women of rural residence had higher prevalence of the contraceptive use and maternal health services except for facility delivery. Modern contraceptive use did not vary significantly by occupational groups (*p* > 0.05), however, was higher among those in the highest wealth quintile (*p* < 0.05), had 1–2 children (*p* < 0.05), not read newspaper (*p* < 0.05), listened to radio(*p* < 0.05), and watched TV(*p* < 0.05). Similar patterns were observed for ANC and facility delivery as well across the wealth and media use variables. Overall, the prevalence of all four outcomes was generally higher for higher wealth quintile, lower parity, and utilisation of radio and TV.

### N.B. *p*-values are calculate from chi-square tests

**Fig. 1:**
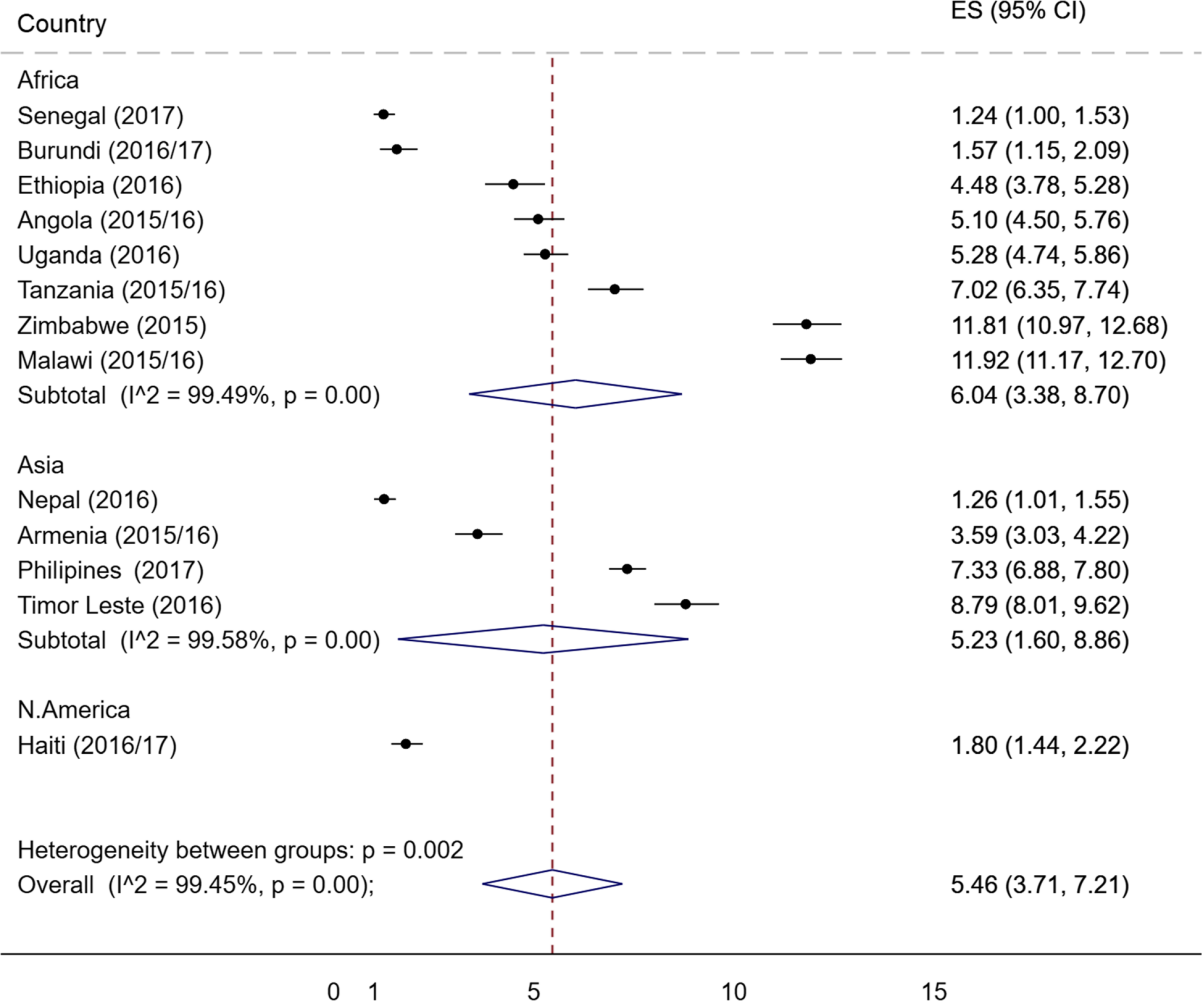
Percentage of women who received SMS about FP. N.B. I-squared statistic (variation in percentages attributable to heterogeneity) indicated high heterogeneity in the prevalence among the countries. (Test for heterogeneity between sub-groups is likely to be invalid for N. America)

Figure 1 shows the results of meta-analysis on the prevalence of receiving SMS about FP in the individual countries. The average for the pooled sample was 5.4% (3.71, 7.21), and was slightly higher in Africa (6.04, 95%CI = 3.38, 8.70) compared with Asia (5.23, 95%CI = 1.60, 8.86). Among the countries from sub-Saharan Africa, Malawi (11.92, 95%CI = 11.17, 12.70) had the highest percent of receiving SMS while Senegal (1.24, 95%CI = 1.00, 1.53) had the lowest.

**Fig. 2:**
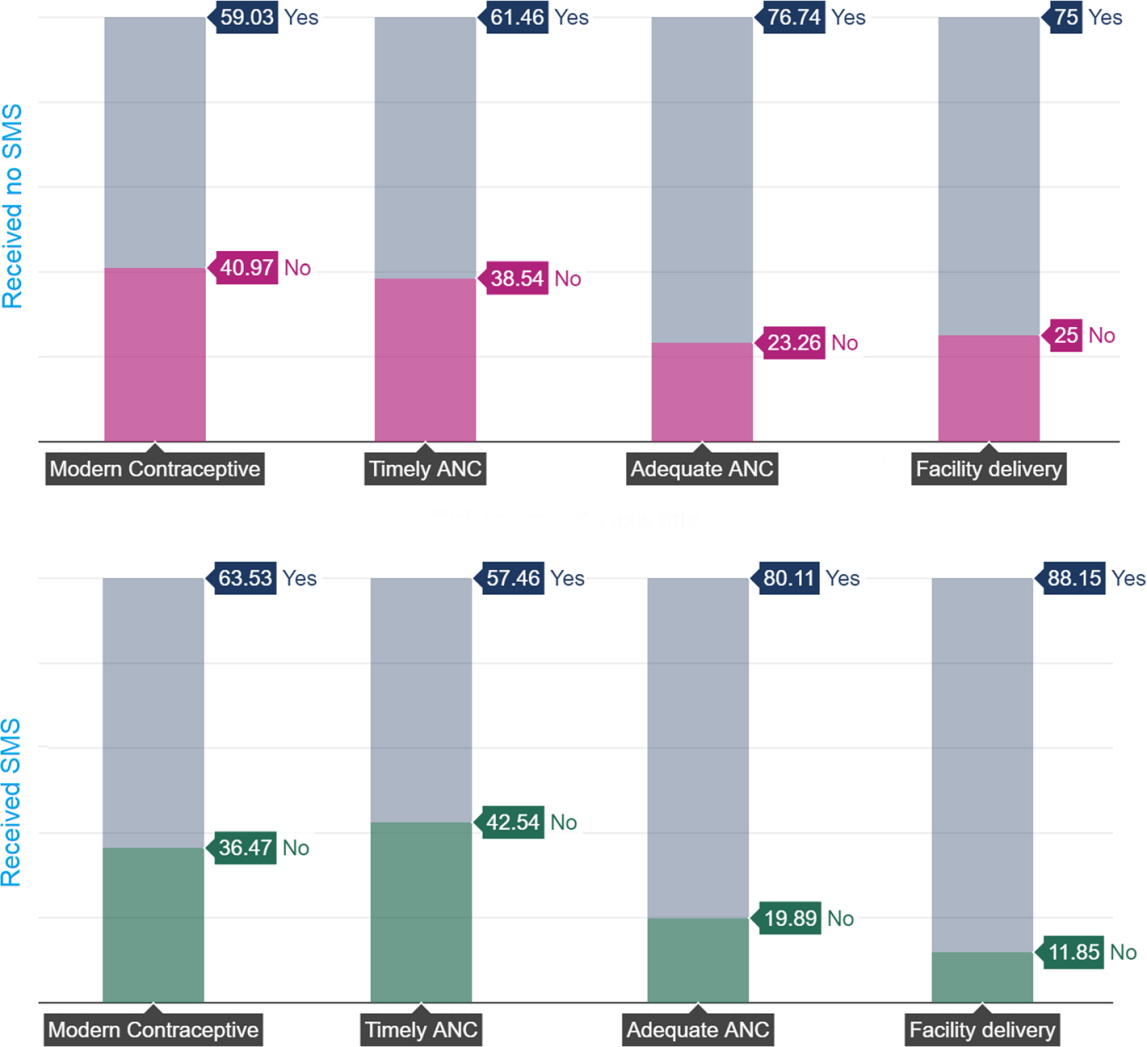
Prevalence of modern contraceptive and maternal health services among those who did and did not receive SMS about FP

Figure 2 illustrates the relative prevalence of modern contraceptive, timely initiation of ANC, adequate ANC and facility delivery among those who did and did not receive SMS about FP. It was clear that the relative percentage of all the outcomes (except timely ANC) was higher among those who reported receiving SMS compared with those who did not.

### Multivariate analysis

Table 3 shows that receiving FP related SMS was associated with higher odds of modern contraceptive use and maternal health services utilisation in the univariate analysis (Models 1). However, the effects for all four outcomes diminished with gradual addition of the covariates (model 2 to 5) such that in the fully adjusted model the association was significant for facility delivery only.

**Table 3:** Odds ratios (95%CIs) of the association between receiving FP related SMS and maternal health services utilisation

	Contraceptive use	Timely ANC	Adequate ANC	Facility delivery
**Model 1**	1.103 [1.041,1.169]	0.883 [0.818,0.954]	1.368 [1.247,1.500]	2.566 [2.294,2.870]
**Model 2**	1.118 [1.056,1.184]	0.861 [0.797,0.930]	1.312 [1.196,1.440]	2.441 [2.178,2.735]
**Model 3**	1.107 [1.044,1.174]	0.875 [0.717,0.939]	1.010 [0.918,1.111]	1.601 [1.421,1.805]
**Model 4**	1.012 [0.954,1.073]	0.967 [0.808,1.331]	1.051 [0.893,1.183]	1.537 [1.363,1.732]
**Model 5**	1.003 [0.946,1.063]	0.975 [0.815,1.140]	0.988 [0.897,1.089]	1.545 [1.370,1.742]

N.B. Model 1: Univariate; Model 2: Adjusted for Age, Residency, Country; Model 3: Adjusted for Model 2 + Wealth quintile, Occupation, Model 4: Adjusted for Model 3, Newspaper, Radio, TV; Model 5: Adjusted for Model 4 + Parity

**Fig. 3:**
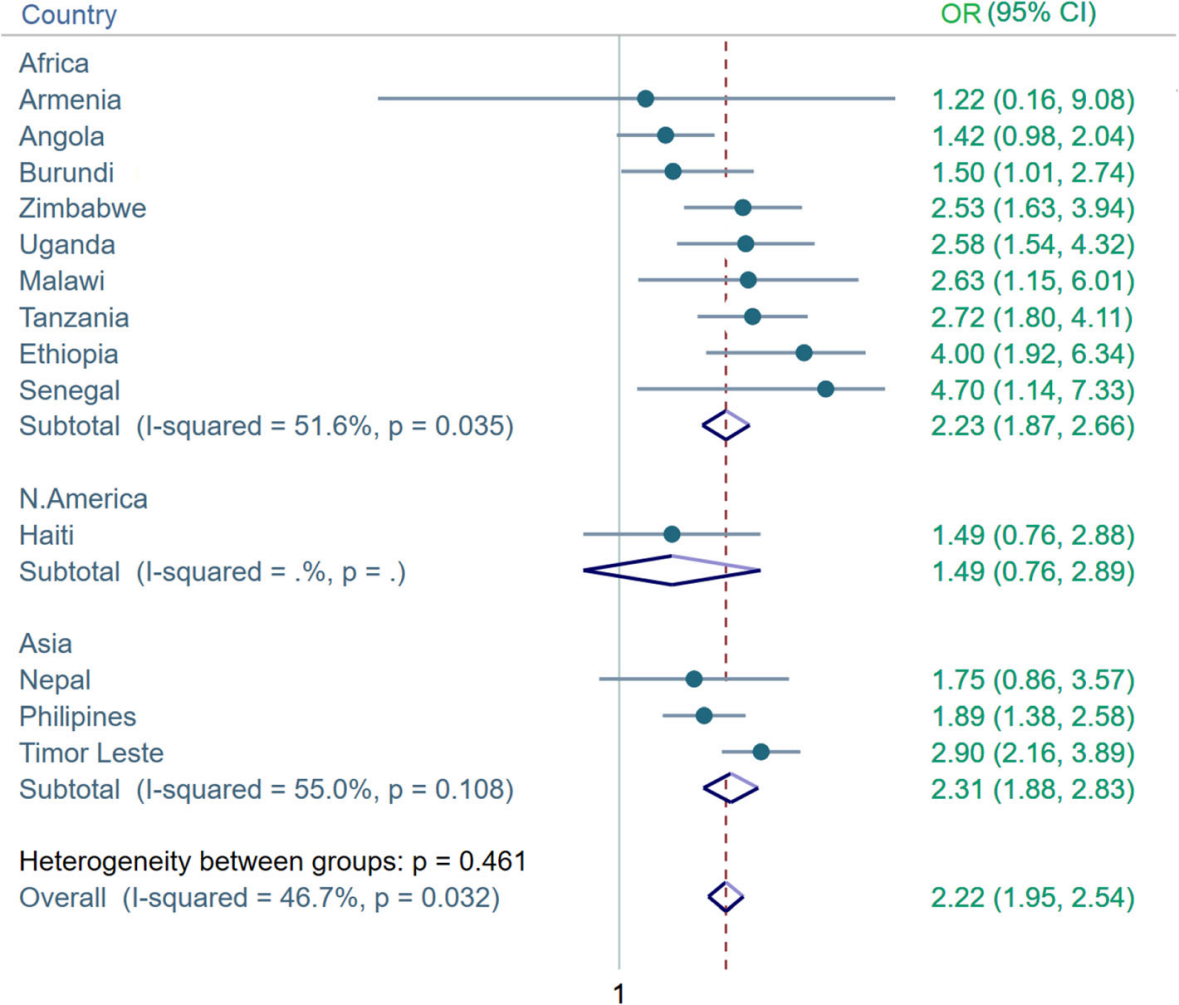
Country-stratified analysis of the association between SMS-based FP communication and use of facility delivery services. N.B. I-squared statistic indicated moderate heterogeneity among the Asian and African countries. (Test for heterogeneity between sub-groups is likely to be invalid for N. America)

Given that finding of the positive association between receiving SMS and facility delivery in the multivariate analysis, we further stratified the analysis by countries and presented the individual association through meta-analysis (Fig. 3). The figure suggests that the pooled odds ratio of having facility delivery was 2.22 (95%CI = 1.95, 2.83) times higher among those who received SMS compared with those who did not. The effect size was highest for Senegal (*OR* = 4.70, 95%CI = 1.14, 7.33) and lowest for Burundi (*OR* = 1.5; 95CI = 1.01, 2.74).

## Discussion

### Main findings

Mobiles phone-based communication are gaining increasing popularity in the LMICs and is being leveraged by the healthcare providers as a cost-effective approach to deliver essential family planning and reproductive counselling. Research based evidence on the relationship between text message communication and reproductive and maternal healthcare utilisation can be extremely useful for healthcare systems in LMICs. From this perspective, the present analysis on 14 countries in Asia and sub-Saharan Africa including Haiti has several important insights to report. Firstly, the extent of SMS-based communication on FP was remarkably low and varied substantially across countries. Only Malawi and Zimbabwe had a coverage of over 10%, whereas the coverage in Burundi, Nepal, and Senegal was below 2%. The combined coverage for nine African countries (6%) was slightly higher than that in four countries in Asia (5.2%). These statistics suggest that mHealth communication remains an almost untapped opportunity for investing in reproductive health in these countries that are generally characterised by high maternal and child mortality and low utilisation of maternal health services. According to the reports of some large-scale population based studies, majority of the countries in sub-Saharan Africa have low maternal healthcare utilisation rates [40–42]. Some of the countries also have very high proportion of unmet need for contraception e.g. Burundi (54.3%) [43] and Nepal (52%) [44]. Countries with similarly low rates of contraception and maternal health services use have been implementing mobiles-based solutions to promote the utilisation of maternal health services by addressing infrastructural and skilled human resource constraints [45–48].

Regarding the association between SMS-communication with modern contraception, only place of delivery was found to be significantly associated. This finding is quite contrary to expectation as women who are exposed to such communication platforms are more likely to adhere to contraceptive use [49–53]. However, one recent systematic review study concluded that the present evidence base is unsubstantial to support large-scale mobile phone-based interventions, and that mobile phone-based service delivery should be considered as part of the wider health service delivery [49]. In this respect, our findings are in line with the current knowledge base, and calls for more in-depth studies to investigate the suboptimal efficacy of the SMS-based interventions for promoting the use of FP methods.

Although our findings do not suggest any positive role of SMS communication, we did observe a strong association for facility-based childbirth. This finding is a unique contribution of our study and opens a new window of research regarding the role that FP communication can play in improving the utilisation of skilled birth services. This finding is worthy of attention for further research and policy attention in the countries with low prevalence of health facility delivery, especially among the marginalised communities. Financial constraint is a key determinant of underutilisation of maternal health services [10, 33, 40, 54] to which mobile-based counselling can provide a cost-effective solution [35, 55]. The wealth gradient in the uptake of maternal health services is also evident from the present findings, as the prevalence of facility delivery was about three-times as high in the highest wealth quintile compared with the lowest. Improving use of family planning and maternal health services is the key to achieving maternal and child health related SDGs in Africa [5]. A sustainable solution to this persisting problem will have to address the deep-rooted socioeconomic and geographic disparities, which might be achieved to a great extent through proper exploitation of mobile phone-based technologies in the coming days.

### Policy implications

Our findings have important policy implications for family planning and maternal healthcare promotion programmes in the countries being studied, and hopefully in other LMICs. Apart from the low coverage of SMS, the descriptive and multivariate analyses revealed significant heterogeneity in cross-country coverage of provision of FP related SMS as well its association with health facility delivery. Countries that are lagging behind in achieving the maternal health related goals may take note of the contrasting scenario and develop strategies to improve the coverage of mobile phone-based FP services. That there was no significant difference in the use of modern contraception in relation to exposure to SMS is a finding of particular concern. Although we did not have any contextual data to assess the source of the insensitivity to SMS communication, this find warrants a deeper investigation into the quality of the services and design of the overall framework. In addition, previous studies have generally attempted to assess the role of SMS based intervention on a particular aspect of family planning or maternal health services. Our findings extend the understanding of the broader role that family planning communications can play on the uptake of maternal health services. It is assumable that women with better knowledge of their contraceptive method are also more aware of antenatal services to ensure a healthy pregnancy and birth outcomes. Although we did not find any significant association between receiving SMS and antenatal visits, the findings support the assumption that that SMS can be an effective intervention for promoting facility delivery services. A recent study based in India reported that integrating family planning with maternal health services can increase postpartum use modern contraceptive [56]. In contrast, our findings suggest integrating family planning communications with maternal health services can improve the utlisation of facility delivery services.

### Strengths and limitations

We have several important strengths and limitations to declare. Based on the nationally representative data from fourteen Demographic and Health Surveys, this was the first multicountry study to report the scenario of SMS based family planning communication and its association with contraceptive use and three key indicators of maternal health utilisation. Almost all the surveys were carried out within a similar time span (2015 and 2016), which meant that the time-lapse effect was not a big issue in the comparability of the findings across countries. We also applied rigorous selection criteria to ensure the validity of the findings. A small proportion of participants reported owing mobile phones and receiving SMS about family planning, and were consequently removed from the analysis for the sake of maintaining logical consistency. While the possibility of receiving SMS on participant’s husband/someone else’s device cannot be ignored, considering so would as well decrease the efficacy of the communication owing to potential misinterpretation of the messages by the third party. Application of such selection criteria had certainly compromised the generalisability of the data, however in so choosing we were able to assess the effect of first-hand communication between participants and the SMS providers. An important limitation in the same context was that we were unable to decipher the content of the SMS, at what frequency they were communicated, and who the providers were (e.g. public or private providers, NGOs). Having details on these factors would help us better understand and interpret the current findings. For instance, the absence of association between receiving SMS and the use of modern contraceptive is a potentially controversial finding whose explanations may lie, at least in part, in the design of the communication services. For the discussion sections, the terms sub-Saharan Africa and Asia should be read cautiously as they refer to the countries covered by this study only.

Another important selection criterion was based on the educational status of the participants. We eliminated the observations with no educational experience as not being able to read is most likely to have no beneficial effect of the SMS communication on contraceptive behaviour and the use of maternal healthcare services. However, we did not keep the education variable in the analysis to avoid multicollinearity issues, as educational status was significantly correlated with household wealth quintile and occupation of the participants. It is also important to note that the prevalence of maternal healthcare service use (timely and adequate use of ANC and facility delivery) may not represent the actual scenario due to the application of these selection criteria.

Lastly, the data were secondary and cross-sectional in nature. Data being secondary meant that we had no control over the selection procedure and accuracy of measuring the variables. Most of the variables were self-reported, and hence remains subject to reporter and recall bias. Data being cross-sectional meant that we are unable to measure the causality or directionality of the associations. This is particularly so for the studies of this kind owing to the inherent difficulty in distinguishing the statistically significant differences from clinically meaningful associations. However, in the context of our study it is arguable that directionality of the association was not a big confounder as receiving SMS is likely to enhance the utilisation of care, however, the reverse may not necessarily be the case.

## Conclusion

The present study reveals significant heterogeneity in cross-country coverage of provision of family planning related SMS, and its association with health facility delivery. A more noticeable finding was the absence of association between SMS-based family planning communication and use of modern contraception. Overall, we found a significant increase in the odds of availing health facility delivery services among those who receive SMS about family planning. However, this was a cross-sectional study and the association needs to be interpreted with caution. In conclusion, the findings help extend the understanding of the roles that SMS-based family planning family planning communications can play on using modern contraceptive methods and uptake of maternal healthcare services.

## Data Availability

Data are available from Demographic and Health Survey website **(**https://dhsprogram.com/data/available-datasets.cfm).

## Abbreviations

ICF: Inner City Fund
LMICs: Low-middle-income countries
